# An Approach to the Unified Conceptualization, Definition, and Characterization of Social Resilience

**DOI:** 10.3390/ijerph19095746

**Published:** 2022-05-09

**Authors:** Jorge Moya, María Goenechea

**Affiliations:** 1 Facultad de Ciencias Económicas, Universidad Autónoma de Madrid, 28049 Madrid, Spain; 2 Facultad de Derecho, Empresa y Gobierno, Universidad Francisco de Vitoria, 28223 Madrid, Spain; maria.goenechea@ufv.es

**Keywords:** social resilience, sustainable development, social capital, resilience policies, adaptive capacity

## Abstract

The purpose of this article is to offer a synthesis of the characteristics of social resilience, integrating the different approaches received from the social sciences. We propose to focus this conceptual framework as a previous and necessary step for the later study of the possible ways of promotion of this social resilience, that will help to strengthen the welfare and public health systems. The paper explores the difficulties in defining these characteristics, identifying their constituent elements. After this, the paper study the challenges to the future development of resilience models, showing the ways that offer some advances. Finally, we conclude that the social resilience must be conceived as a dynamic, multi-level, and evolutionary process if we are to help societies not only cope with adversity but also to adapt and transform themselves.

## 1. Introduction and Methodology

The concept of resilience is now predominately related to the study of how individuals and groups respond to adversity, dealing with shocks and disturbances to adapt and evolve to return to a former state of equilibrium. The notion is useful in analyzing why certain individuals and groups respond better than others to adverse circumstances and has become a key characteristic in the sustainability and socio–health progress of both individuals and whole societies. Resilience refers to the ability to successfully overcome challenges and deal with changes, which offer experience and opportunities for learning and growth. Its development at the community level has very important implications for the public health variables and the health care services of individuals and societies [1,2].

The relationship between social resilience and public health runs in both directions. On the one hand, improving social resilience offers value to public health in coping with illness, and under emergency scenarios [3]. On the other hand, the daily practice of the professionals of public health services can be guided by practical programs that, similar to those that promote the safety and the general health of the population, improve the resilience of the community. The main support of this social resilience to the public health systems has traditionally focused on preparing our societies for the arrival of disasters, and on the risk reductions. However, recently, policies are beginning to focus their interests on the assets that societies already have, for strengthening these assets and reducing the threats and costs of the public health services in the long term [4,5]. The development of social and family support networks, the policies focused on improving social cohesion, the promotion of social services for the generation of interpersonal connections, and other participation in organizations connected to the public health services are some examples of this approach where social well-being is linked with public health [3,6,7].

The promotion of this social resilience has, in many cases, become a goal of the government agencies associated to the public health systems. In the United States, the 21st Homeland Security Presidential Directive of 2008 [8] had already identified the social resilience as one of the four critical components for the public health and medical care. In the same country, community resilience appeared in the National Health Security Strategy of 2009, and in the National Disaster Recovery Framework of 2010, to repeat its presence in the Presidential Policy Directive of 2011. In the following years, the importance of social resilience for safety and health has been increasingly recognized [2,5,9].

However, the current challenge is still to know how this social resilience can be developed, which programs should be used in public health, and how to measure their results. Due to the multifaceted nature of the concept and the large number of multilevel variables involved in this social resilience, it is not easy to implement practices that support its development [10]. The basic problem for this is that it is necessary to achieve a consensus on a previous definition and theoretical conceptualization that will allow the further implementation of applied public health programs that really increase the social resilience [11]. This lack of definition does not allow identifying and distinguishing the assets that truly generate community resilience and not simply social well-being. We have to distinguish a generally well-prepared society from a resilient society. For this, the conceptualization on social resilience would be the first step to later studying the processes that will help for developing it [12,13], and this is the purpose of this paper.

Although the original term comes from materials engineering studies, from the 1970s it began to be used also in ecology and psychology, and finally it has since been expanded to the study of complex adaptive systems more generally [14]. In recent years, the term has served to offer an approach towards understanding social dynamics in the event of stresses caused by political, social, or economic change. Thus, the concept has gradually extended its reach to refer not only to individual resilience but social resilience, more broadly in the understanding processes of human adaptation [15] and the advantages and opportunities change may bring [16,17,18,19], always in connection with the sociosanitary improvements resulting from its improvement [4].

The use of this term has grown in recent decades and there are several disciplines that use it, so there is a certain diversity of approaches associated with this initial definition [20]. Up to thirty different definitions have been made for the term resilience [21]. For this reason, this paper examines the characteristics that this social resilience must have in order to become a useful tool for the management of social change. For this purpose, this article offers a rigorous review of those articles dealing with the concept of social resilience in order to analyze the state of the studies published about this issue, specifically regarding the concept of resilience, the factors that increase it, the relationship between individual and social resilience, and examples of models of resilience.

The methodology for the study was conducted through several steps. As a first step, when we became interested in this issue, we analyzed some articles and studied their references, identifying twenty papers widely cited in these articles published in recent years. They were considered important papers on the concept and characterization of the term social resilience.

In order to delve deeper into the study of this issue, a search was then conducted on the Journal of Citations Reports (JCR) database for the term “social resilience” in the last decade. The results were cross-referenced with these initial twenty selected articles, twelve of which were found to appear in the search results. The other eight articles were added to the sample for a total of 583 references.

The next step consisted of reading the abstracts of those articles to eliminate those which had a different focus, often referring exclusively to the fields of individual psychology or natural ecology. Successive filters were applied to narrow the search, reduced exclusively to articles centered on the definition and characterization of social resilience. The final result was a total of 151 useful references which were then analyzed in detail, and a summary of their content allowed for the elimination of articles which were very closely matched or repetitive. Finally, the review found 98 references that were classified according to the main focus of the papers.

Finally, only 51 of them have been included in this paper, among other references. They are presented in Table 1, organized by the main focus offered in each of these papers.

**Figure 1:**
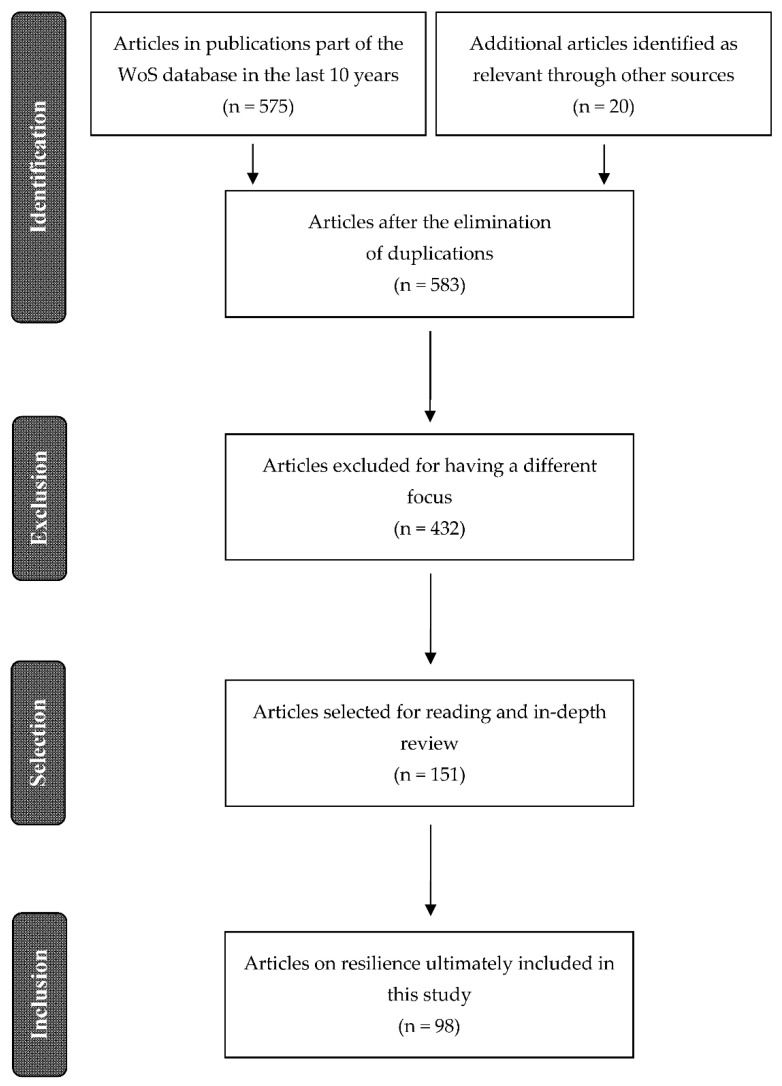
PRISMA flowchart of the bibliographical selection process.

A PRISMA flowchart [72] of this revision process is provided in Figure 1.

This was the total number of articles examined in detail in order to extract the elements of social resilience that allow us to establish the defining characteristics of the concept. The interest of this study is precisely to focus the current dispersion of approaches to social resilience by achieving a consensus on the main characteristics of the notion. Thus, in the future, it will be easier to seek improvements in the resilience of societies. This would be difficult to achieve without knowing exactly what characteristics and elements are involved in its definition.

## 2. Origin, Definition, and Evolution of the Term “Resilience”

The word “resilience” originates from the Latin “resalire”, meaning “to spring back” or “rebound”. Although many studies point out that the notion of resilience was initially introduced by C. S. Holling in a paper of 1973 on Ecology [73], it has been shown that the term originally comes from the studies of strength of materials [20,74,75] (Alexander 2013; Brown and Williams, 2015; Pushpalal and Suzuki 2020). Here, in engineering, resilience refers to the capacity of materials to return to their original form when subjected to certain external forces [76,77] Therefore, resilience basically focuses here on the elasticity of materials, beyond the study of the classic properties of resistance of materials, determining the point beyond which materials or systems will be unable to return to their original state [78]. In ecology, the term is used for referring to the capacity of an ecosystem to sustain itself and recover after disruptions or disturbances. The coining of this term led to numerous subsequent works in the field of ecology [79] focused on the manner in which the ecosystems absorb or adapt to alterations (earthquakes, fire, floods, infestations, and human-induced alterations of the environment). Thus, resilience was understood as the capacity to react to changes while maintaining the essential elements of an original state [22,24,80]. In its sense, resilience was associated with the capacity to resist external shocks [81], and the capacity to overcome, recover, and adapt [82]. In the field of ecology, the term resilience has improved the term sustainability, which refers to the capacity of an ecosystem to perpetuate itself, but resilience goes further, considering the notion of adaptability; that is, considering the disturbances or changes an ecosystem may absorb before being entirely reconstituted in a manner completely different from its original state.

The term resilience was adopted in psychology. Here, the concept of resilience arose from studies on children who did not develop psychological problems despite being exposed to conditions which were predicted to lead to psychological disorders. Here, resilience is referred to as an individual’s capacity to adapt, not only for resisting the adversity by invulnerability, even leading to greater strength and fortitude [83]. Studies into psychologically resistant character traits were followed by further studies based on the interaction between an individual and their environment which favors healthy psychological adaptation [7,84,85,86]. Although the term would become further nuanced in the field of psychology [87], certain traces of its original meaning within the field of ecology persist. The latest extension of the term has entered to field of urban planning and development, where the conceptualization of the city as a social ecosystem raises the notion of creating resilient spaces [88,89,90] able to endure future changes, particularly in the face of climate change.

In each of these three fields, ecology, engineering, and psychology, the notion of resilience offered greater scope for understanding than the terms previously used in these fields. In ecology, the notion of resilience goes beyond sustainability, referring to the disturbances an ecosystem can withstand before being entirely reconstituted in a manner completely different from its original state. This is similar to developments in the field of psychology, where the term “resilience” superseded the notion of invulnerability, given that the former could be developed within the individual [91] while invulnerability was considered as an intrinsic trait of the agent [92]. Finally, in engineering, resilience focuses on the properties of elasticity of a specific material, enabling it to be deformed and return to its original state [80] differentiated from the classic properties of resistance of materials, focusing on the capacity of materials to resist compression, tension, torsion, or sheer [78].

## 3. From Individual Resilience to Social Resilience

After engineering, the ecological focus of the notion of resilience was long predominant, but the advance of the research in social sciences incorporated a new relative notion, called socioecological resilience [93,94]. Of course, with this approach, the initial ecological viewpoint remained prominent while incorporating other aspects of a sanitary and social nature [18,95,96] related to resilience. Of course, is possible to find an overlap in the concepts, perspectives, and focus. For example, there is important academic literature on disaster resilience, focusing on responses of social structures to disasters or natural hazards, with connections with the social–ecological resilience [97].

The study of the way in which individuals overcome the tensions and stresses of adversity and traumatic events demonstrated through this new socioecological focus of resilience that certain networks or resources within one’s environment, such as social or cultural, serve as support and help build the capacity for resilience within individuals. Logically, the individual does not exist alone, but rather within a community which offers resources which may serve to strengthen the resilience of the individual [30,52,98], based on relations and values such as inclusion, trust, mutual support, or cooperation [99]. This set of values for social support constitute something that in sociology is referred to as social capital, which was soon associated with the development of resilience [56,71]. This inclusion of environmental or social elements, constituting social capital, should not be confused with social resilience. There is no doubt that social capital can lead to the development of social resilience [53,69], and a number of authors have signaled that both capacities may be mutually reinforcing [23,29]. However, the relation between the two concepts is more complex and less linear than they may appear [57]. In any case, the notion of individual resilience gradually extends to that of social resilience [100] via the intermediate concept we may call “socioecological resilience” [40,41,48,59,101], which incorporates aspects of this social capital.

From the review of the literature, it is important to understand that social resilience is not simply an aggregation of resilient individuals, but the manner in which a community responds, as a whole, to stresses and tensions that impact them as a group [65], also that it is an adaptive phenomenon. Depending on the resilience not only of individuals but of the community and its social systems, the community can come up with responses to disturbances, adapting and evolving in a process that may give rise to new opportunities for change without renouncing the core essence of its origins [102]. A true understanding of social resilience involves considering it as a dynamic [103] and multi-disciplinary process [20,23,60,68,104], with a multitude of interdependent aspects (economic, psychological, social, etc.) and levels (for example, between individuals and their socioeconomic environment) [31,51,105]. To date, there has been little research with this multi-level approach to analysis [106]. This is largely because this type of analysis requires an understanding of how social resilience emerges from the individual resilience, identifying those aspects which facilitate this process. It is precisely this linkage which has received scant analysis [102].

The possible interactions of all these processes, at different levels, contributes to the multiplication of components which determine social resilience. A multi-level approach increases the number of possible strategies of adaptation, resistance, and transformation to deal with shocks and disturbances [42], making it difficult to establish the precise relationship between individual resilience and social resilience [26,32,49,64]. There is no doubt that studies into individual resilience can offer clues as to the elements which may further the development of strengths within the entire society, and vice versa [68], achieving public health improvements. This leads to the next section of this work, which finally explores the features that characterize social resilience.

## 4. Principal Characteristic of Social Resilience

There are many characteristics and elements that appear in the articles studied on social resilience. Of course, it is interesting to group them meaningfully in order to offer solutions in their conceptualization. A great deal of the examined literature refers to three principal characteristics of social resilience [28,92,93,107,108]. These are the same terms referred to by the Resilience Alliance, a multidisciplinary association for the study of socioecological resilience. Although terminology varies, these are habitually designated as resistance, adaptability, and transformability. In fact, these three aspects of resilience largely concur with the chronological development of the term and study into resilience.

The first of these characteristics, resistance, has also been expressed as persistence [69,73,81] and refers to the degree of change or transformation a complex system can withstand while preserving its functional and structural characteristics. This is not simply the stability of the system, nor the capacity to return to a previous state of equilibrium, but rather refers to the capacity of resistance of the system itself to disturbances before reaching the point where its principal features, functions, and processes collapse.

The second characteristic of resilience, adaptability, refers to the capacity of a system to absorb the disturbances while retaining those features which are part of its original identity [95]. Adaptability is not the capacity for adapting to change, but the capacity for absorbing the changes. This was primarily the focus of so-called socioecological resilience, highlighting the capacity of agents to learn from experience, accumulate knowledge, and respond by adapting to changing conditions. Adaptability thus refers to the possibilities of an individual or system to redesign its structures, its capacity for self-organization and reorganization to meet the challenges of external disturbances and change [19,62,82,109,110]. The notion of adaptability emphasizes the evolutional or dynamic connotations of resilience rather than the idea of returning to a prior state of equilibrium [96,111,112,113].

Finally, the third characteristic of social resilience extracted from the study of all the academic papers is transformability. It refers to the capacity of a system to evolve from its original state towards alternatives which are compatible with survival under new conditions [17,81]. Here, it is implicitly assumed that stresses and disturbances may make the exact original system unsustainable and there are a multitude of potential states into which the society may evolve and mutate. This transformability should be understood as functional, referring to the structure of the system itself. Social resilience includes, as it has been said, the capacity of a society to adapt to change, learning and acquiring the right knowledge and capabilities to address new challenges, now transforming its internal dynamics in ways that ensure survival and sustainability [18,82,111,113,114]. This is an important aspect of the social resilience because it has attracted the interest of researchers in determining the learning capacity of a social system [15,115,116,117,118,119].

**Figure 2:**
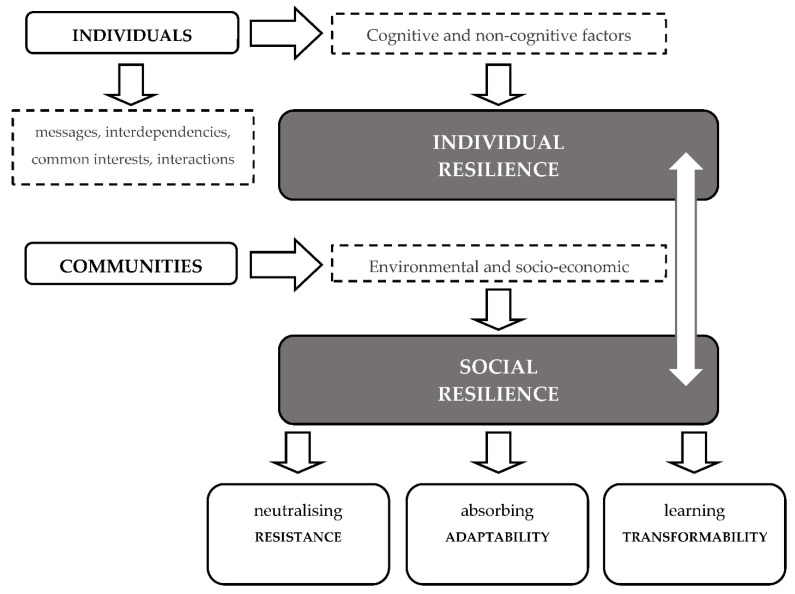
Relationship diagram and characteristics process. Each of these three characteristics also have a specific temporal frame of reference. The first, resistance, the capacity to withstand change, is strongly rooted in the past; adaptability refers to the present while transformability is oriented towards the future of the system and society. These aspects are summarized in Table 2.

Then, the true social resilience should involve these three characteristics [28], from the mitigation of disturbances (resistance) to the reorganization of the system to absorb the external shocks (adaptability) and the development of learning ways for allowing for systemic transformation in the face of new conditions (transformability). The integration of these three characteristics points towards a more precise definition of resilience. Therefore, this social resilience is now the capacity of a system, society, or group to resist and absorb the hazards, preserving its essential basic structures and transforming itself in a manner that better ensures it survival in the future. This characterization of resilience assumes a dynamic process, as noted above, and not merely a specific trait that facilitates the stability [82,111,113]. The Figure 2 provides a useful overview.

## 5. First Steps for Developing Social Resilience

There are many studies on resilience discussing policies implemented in different sectors, but they offer, in the majority of the cases, very specific and partial results that cannot properly consolidate explanations that allow the development of global models. However, there are groups of studies using applied work on social resilience that allow to show some trends. From the study of the most relevant bibliography about this, two main groups of works have been found. On the one hand, there are interventions oriented towards sustaining and amplifying the emotional factors through social resilience strategies based on interrelationships between the actors. On the other hand, there are policies for the governance of resilient communities based on the promotion of the social learning and on the development of resilient social capital. Both lines are important for promoting socio–health improvements in the society as a whole. Note that the first way is focused on resistance (reactive), while the second way points towards the adaptability and the transformability (proactive), following the three characteristics described above (Obrist et al., 2010).

In the first point, on policies based on emotional factors, it is important to see that operational interventions usually focus on the capacity to promote the so-called social networks of supporters. These structures for supporting the individuals determine the emotional sustainability of the agents in a society [58]. These networks are, in a general sense, all the networks that promote trusting relationships in a society, such as friendship or family [120]. The density of the social interactions between the members of this kind of groups has a positive influence for the social resilience [43]. These social support networks favor the social cohesion through the establishment of strong relationships. Support from family and friends has been shown to be a significant predictor of a society’s resilience [39,50,52]. These supportive relationships between the members of a family generate social resilience, especially in the case of unemployed people [44,54]. Some papers have referred to this support to help to overcome tensions in the family level [46], or in the case of medical problems [47], or with complicated states typical of the elderly, helping them by having inclusive community organizations [70,121]. The resilience of young people increases when they are feeling supported by their social and family environment [45,122]. In general, the quality of relationships between the individual and the social groups is an important point because this increases the chances of the policies becoming successful [36], which should be taken into consideration in the health provision programs.

In addition, the moderating role of the conciliatory elements in the field of the relationships, even in scenarios where a situation of prolonged conflict has occurred, help in the development of the community resilience [25]. On the contrary, factors that contribute to the deterioration of relationships between individuals, such as violence, insecurity, or social confrontation, are a threat for the individual well-being and they are the main destructive element of the social resilience.

Finally, concerning the importance of the relationships and the social support networks for the development of social resilience, we emphasize two points. First, that social support is more conducive to the development of resilience in people than those other individual factors that, such as self-esteem, can be stimulated by the agents themselves [67], and, secondly, that this social and family support can also be promoted ex-post, for instance, in the necessary recovery after a disaster [123].

The second category cited on applied research on social resilience is connected with the promotion of the knowledge and the social learning of the capacities of transformation in a social innovation context. This is encouraged by the promotion of an executive social intelligence and a target-oriented mentality towards the transformation. The main point here is called the social memory, which helps to establish pathways for the development of this community resilience. The idea is to understand how the past has influenced the knowledge that we have today, in order to provide answers to the current risks that will promote a social resilience [66], improving the socio–health level of a society. This includes having a previous knowledge of the weaknesses of a society before potential disasters [124]. On the contrary, members of a society that are not prepared by their past to understand the new problems will live in a state of continuous threat and uncertainty that gradually undermines their capacity for resilience [61]. It is possible to improve the social resilience in this direction also by the civic participation [55] by the strengthening of the relationships among the social community and its institutions [6]. The collaborative approaches may be the most appropriate ways to promote the social and institutional learning for this kind of transformational social resilience, as several studies have shown [7,11,125].

This social resilience which is acquired through knowledge and social learning can be obtained also by the communication of the risks that a society may face, for a correct perception of them, in contrast with a sweet ignorance of these potential risks [7]. Of course, during a disaster it is mandatory to provide the best information to the population about the relief works, possible help-points, and the consequences of the impacts received. However, here, the studies refer to the provision of information in advance, also in the health service units [126]. These strategies for communicating potential risks allow the incorporation of the information, helping to achieve the conceptualization and internalization of the potential impacts, improving social resilience [34]. On the contrary, the ignorance of the risks generates uncertainty and a low responsiveness in the people and in the societies.

These policies create an executive social intelligence that, in addition with the promotion of the individual adaptive capacity, provides, at the social level, the cognitive and emotional tools needed to enhance social resilience [33]. In general, the governance for building this social resilience is not easily compatible with policies of rigid responses to the changes. The reason for this is that the construction of social resilience is a process of gradual assimilation of information, where the flexibility allows the emergence of progressive elements of resilience that will help the control and the governance [127]. Studies suggest that this social resilience can also be increased by ways of meta-flexibility in dynamics of groups, by flexible positions that allow movements from a state to another state, as the best and most appropriate response in complicated scenarios [27].

Of course, the review of the cited literature shows that there are more studies on specific applied policies in some sectors, but they do not offer specific results, making impossible the establishment of relationships that would allow us to build general models for the development of the social resilience and the improvement of public health systems. It is necessary to wait for further studies that, in the future, are able to provide more consolidated policies.

## 6. Conclusions and Suggestions for the Future Research into Social Resilience

The majority of articles on resilience have focused on those factors which promote individual resilience, especially researching in the psychosocial and neurobiological elements. However, regarding social resilience, while the research has intuited that it will be a key element in the future development of the health and welfare of our societies, the novelty of this construct is so recent that it has been difficult to properly concrete its real nature and depth. The main purpose of this article is to contribute to reaching a consensus on a definitive definition based on previous research, which will allow the generation of models for its development.

Related to this first problem of general lack of definition, the majority of the studies have been focused on specific problems, using the operativity of this social resilience, but they have not performed a complete theorization of the concept. The reason is because, in these studies, there are not many valid conclusions for the development of an applied technique or model for the improvement of this social resilience. Therefore, the studies on social resilience have generally failed to bridge the gap between theoretical research and practical, and applicable models do not exist [60]. The problem is that social resilience depends on a multitude of often disparate variables that do not allow themselves concrete or practical interventions. Of course, it is important to identify how the alteration of certain variables related to this resilience have implications in other variables, and in the public health of the community, but the problem is not only to establish the relation between the variables. It is necessary to measure not only the impact that a specific problem with one variable will have on other variables, but mainly on the adaptive capacity of an entire community and its welfare, too [38]. Therefore, the problems, ultimately, impact not only the directly connected variables, but all other variables and levels as well, gradually undermining the flexibility necessary to adapt or change. This is the real risk for the society and its health system, because the connected and growing problems weaken the flexibility and the capacity for learning and reorganization of the society [35]. Thus, the main obstacle to the growth of resilience and social welfare is the lack of knowledge about the relationships between variables, and the multilevel implications of each of these variables.

In addition, the complexity of the cause–effect dynamics of these processes [49], especially in the relationships between individual resilience and social resilience [64], does not make matters easier. Clearly, there is a feedback loop or bidirectional relation between the two resiliencies, individual and social [26]. The studies into individual resilience can offer insights into the factors which can strengthen, or weaken, the resilience of communities and systems [68]. There remains, however, a great deal of research to be carried out in understanding these relations, and there are not enough works about this point [26].

The main important conclusion is that a deeper understanding of social resilience requires multi-level (individual, group, and social) and multi-factor analysis, which remains lacking. Future research should take a multi-relational approach, incorporating factors associated with the environment and aspects related to the varying levels of association between individuals in the communities. This will permit the development of dynamic models [68] that show the possible relations between the variables and levels, and subsequent study of the specific elements which stimulate the potential of a socio–health system of the society to resist, adapt, or transform itself in the face of adversity [118], with independence of the origin of the disturbances, either economic, political, or environmental [62].

For this, the first part of the challenge is to further develop the conceptual aspects of resilience as we have intended in this work, for incorporating data to better measure the levels of current social resilience [128]. The integration of transversal research will help gain a deeper understanding of the possible natural cycles of adaptation of our societies once the accelerating elements of social resilience have been clearly identified. Just as the aforementioned studies here that have been made to identify these elements, it would be fruitful to conduct an in-depth evaluation of contextual factors such as inequality, uncertainty, violence, the lack of integration and social cohesion, etc., which may impair social resilience [37,63].

The cited difficulties in the understanding of the cause–effect relationships also affect the development of an effective governance system that facilitates the improvement of the social resilience [49,129]. Much work remains to be carried out in creating accurate causal models which can help establish valid guidelines for government health action in boosting social resilience. Of course, these models must also consider the important role of institutions in building healthy and resilient societies and their actions in political, social, sanitary, and economic spheres. It is suggested that it will be an important help to model scenarios using simulation techniques based on communities and agents, as well as global structural models that serve to improve our understanding of social resilience, creating accurate indicators over the factors involved in the growth of the social resilience.

It will be necessary to obtain an adaptive approach to resilience in order to understand the complex dynamics and equilibrium between resistance, adaptation, and transformation. There is no doubt that a minimum degree of stability is necessary to establish the basic outlines of models to identify the sources of adaptability. In terms of social capital, we are far from understanding its true relation to resilience, except in very short-term situations. However, we do know there are certain prior minimum conditions upon which social resilience depends and without which social resilience is impossible. The identification and measurement of these minimum conditions is an inviting field of future research. These conditions can be revealed by the study of societies under chronic conditions of adversity and stress [65] that are failing to develop social resilience.

Finally, practical research in the future must offer models which, accepting the characterization of social resilience and the factors which condition its development and that are provided here, serve as effective tools to facilitate the emergence of more resilient societies.

## Figures and Tables

**Table 1:** Summary of main aspects of resilience analyzed in these articles referred.

Field	Articles Analyzed that Study This Issue
Definition of resilience	[20,22,23,24,25,26,27,28,29,30,31]
Factors that increase resilience	[27,28,29,30,31,32,33,34,35,36,37,38,39,40,41,42,43,44,45,46,47,48,49,50,51,52,53,54,55,56,57,58,59,60,61,62,63]
From individual to social resilience	[58,59,60,64,65,66,67,68]
Models of resilience	[27,29,31,51,52,56,57,59]
Examples of resilience	[29,51,52,53,54,55,56,57,58,61,62,69,70,71]

**Table 2:** Summary of aspects of resilience and its principal characteristics.

Field	Former Focus	Principal Aspects	Principal Focus	Equilibrium
Engineering	Resistance of materials	Time to return, recovery, efficiency	Resistance	Proximity and the return to a state of equilibrium
Ecology	Sustainability	Impact mitigation, maintenance of functions in the face of shock	Resistance Adaptability	Possible alternative equilibriums, stability of the original environment
Psychology	Invulnerability	Overcoming, strength and fortitude in the face of adversity	Resistance	Multiple possible equilibriums in overcoming adversity
Social Resilience	Static resilience on the same level	Durability, capacity to adapt, transformability, learning and innovation	Resistance Adaptability Transformability	Multiple dynamic and evolutionary equilibriums

## Data Availability

Not applicable.
